# *Brchli1* mutation induces bright yellow leaves by disrupting magnesium chelatase I subunit function in Chinese cabbage (*Brassica rapa* L. ssp. *pekinensis*)

**DOI:** 10.3389/fpls.2024.1450242

**Published:** 2024-08-30

**Authors:** Chuanhong Liu, Yi Chai, Chong Tan, Fengyan Shi, Yun Zhang, Zhiyong Liu

**Affiliations:** ^1^ Laboratory of Vegetable Genetics Breeding and Biotechnology, Department of Horticulture, Shenyang Agricultural University, Shenyang, China; ^2^ Vegetable Research Institute, Liaoning Academy of Agricultural Sciences, Shenyang, China

**Keywords:** *Brassica rapa*, bright yellow leaves, MutMap, magnesium chelatase, CHLI, protoporphyrin, chlorophyll biosynthesis

## Abstract

Magnesium chelatase (MgCh) plays a pivotal role in photosynthesis, catalyzing the insertion of magnesium into protoporphyrin IX (Proto IX), a key intermediate in chlorophyll (Chl) biosynthesis. MgCh is a heteromeric complex composed of the MgCh D subunit (CHLD), the MgCh H subunit (CHLH), and the MgCh I subunit (CHLI). The *bright yellow leaves* (*byl*) mutant was obtained through ethyl methanesulfonate (EMS) mutagenesis of the ‘FT’ Chinese cabbage (*Brassica rapa* L. ssp. *pekinensis*) doubled haploid line, whose Chl content, net photosynthetic rate (*Pn*), and non-photochemical quenching coefficient (NPQ) were decreased, and whose chloroplast development was incomplete. *byl* recovered to a light green phenotype under weak light conditions. Genetic analysis revealed that the bright yellow leaves phenotype of *byl* was caused by a single recessive nuclear gene. Using Mutmap sequencing and Kompetitive allele-specific PCR (KASP) identification, *BraA01g010040.3.5C*, encoding the CHLI subunit of MgCh, was identified as the candidate gene and named *Brchli1.* A nonsynonymous G-to-A mutation in the *Brchli1* exon resulted in the substitution of aspartic acid with asparagine. *Brchli1*-silenced Chinese cabbage displayed bright yellow leaves with decreased *Brchli1* expression. Transiently overexpressed *Brchli1* in the *byl* mutant restored the green leaf phenotype and significantly increased relative *Brchli1* expression levels. Both BrCHLI1 and its mutated variant were localized in chloroplasts. Yeast two-hybrid and luciferase complementation imaging assays demonstrated that BrCHLI1 interacted with both BrCHLD and itself. BrCHLI1 mutations did not affect its interaction with BrCHLD. Together, *Brchli1* mutations impaired the function of MgCh, providing insights into the molecular mechanism of leaf coloration.

## Introduction

Chlorophyll (Chl) is a photosynthetic pigment present in plants, algae, and certain bacteria, that plays a pivotal role in photosynthesis (Kunugi et al., 2013). Leaf color is linked to the biosynthesis of heme and Chl, both situated downstream in the tetrapyrrole degradation metabolic pathway. The biosynthetic pathways of Chl and heme both proceed from 5-aminolevulinic acid (ALA) to protoporphyrin IX (Proto IX). The divergence lies in the synthesis of Mg-Proto IX under Mg^2+^ involvement (Brzezowski et al., 2015), leading to Chl production, while heme synthesis occurs with the participation of Fe^2+^ (Masuda, 2008).

Magnesium chelatase (MgCh), a heterotrimer consisting of D, H, and I subunits, is a key enzyme in Chl synthesis found ubiquitously in photosynthetic bacteria and plants (Masuda, 2008). It catalyzes Mg^2+^ insertion into Proto IX, leading to the final synthesis of Chl (Masuda, 2008). MgCh was first discovered to possess catalytic activity in pea (Sirijovski, 2006). Enzyme-catalyzed reactions requiring ATP hydrolysis involve two steps: activation and insertion. The CHLI subunit catalyzes ATP hydrolysis (Reid et al., 2003) and stabilizes the CHLI-CHLD-Mg-ATP complex in the enzymatic reaction (Lake et al., 2004a). Glutamic acid 660 (E660) in the CHLH subunit is crucial for magnesium insertion because it binds the porphyrin in MgCh (Adams et al., 2020). The I and D subunits are members of the AAA+ family of ATPases associated with various cellular activities. They readily form hexamers or heptamers, but notably, the D subunit lacks ATPase activity, whereas the I subunit possesses it (Vale, 2000). The CHLI and CHLD subunits form an AAA+ complex in the presence of Mg^2+^ and ATP, which then interacts with the CHLH-chelating subunit to form an active chelatase (Farmer et al., 2019).

CHLI is the smallest subunit of MgCh, with a molecular weight of 38−45 kDa. It contains the structural domains Sensor I, Sensor II, Walker A, Walker B, and I Rfinger, which are involved in ATP hydrolysis. Walker A stabilizes the binding of ATP, whereas Walker B facilitates ATP hydrolysis by binding to Mg^2+^ (Jessop et al., 2021). In *Arabidopsis* and pea, the semidehydroascorbate in CHLI’s C-terminal helix is easily oxidized, leading to undetectable ATPase activity in oxidizing environments. However, its interaction with thioredoxin (TRX) reductase can restore activity upon adding TRX reducing agents to oxidized CHLI (Kobayashi et al., 2008).

Numerous *CHLI* mutants have been identified that induce changes in leaf coloration. In *Arabidopsis*, insertion of a T nucleotide in *AtCHLI1* resulted in a pale green color mutation (Koncz et al., 1990); the *CHLI2* T-DNA knockout mutant was still able to accumulate a certain amount of Chl (Rissler et al., 2002); *CHLI2* aided in the construction of MgCh complexes (Kobayashi et al., 2008); double knockout mutants of *chli1* and *chli2* exhibited albino leaves (Huang and Li, 2009). In barley, ATPase activity of CHLI was essential for maintaining functionality of the D-subunit (Lake et al., 2004b). Ethyl methanesulfonate (EMS) mutagenesis in cucumber identified a mutant with yellow leaves caused by a *CsChlI* point mutation (Gao et al., 2016). Amino acid missense mutations in *ChlI* resulted in a yellow-green phenotype in rice (Zhang et al., 2006). *TaCHLI* single-nucleotide mutations in wheat were responsible for pale green leaves caused by impaired Chl synthesis (Wang et al., 2020). A mutant variant of *CHLI* with yellow-green leaves was identified in strawberries (Ma et al., 2023). However, the *CHLI* gene has not yet been studied in Chinese cabbage.

Reports indicate that certain plant phenotypes can change in response to variations in light intensity. The *immutans* mutant of *Arabidopsis* showed green leaves under low light conditions, but variegated leaves under high light conditions (Rosso et al., 2009). The Chl-less barley mutant NYB exhibited a yellower leaf color under higher light intensities (Yuan et al., 2010b). In a study on *Acer palmatum*, the ‘Jingling Huangfeng’ mutant displayed a yellow leaf phenotype, with leaves turning green under low light intensity conditions (Li et al., 2015). The R24 photosensitive chlorotic mutant was isolated in pepper, and under shaded low light intensity conditions the Chl content of tea was increased (Liu et al., 2018). The R24 mutant had yellow leaves under low light conditions, but the leaf color turned green and the Chl content increased with increasing light intensity (Yang et al., 2023).

The candidate gene for the golden inner leaf mutant has been identified as *BraA09g007080.3C* in Chinese cabbage (Zhang et al., 2022). Studies on the CHLI subunit in Chinese cabbage have not been reported. Here, we isolated *bright yellow leaves* (*byl*) mutant from an EMS-mutagenized population, which showed impaired chloroplast development and decreased Chl, photosynthetic rate (*Pn*), and non-photochemical quenching (NPQ). Under conditions of low light intensity, the *byl* mutant reverted to a light green phenotype. MutMap and Kompetitive Allele-Specific PCR (KASP) analyses identified *BraA01g010040.3.5C* as the candidate gene, which harbors a non-synonymous point mutation and encodes the CHLI subunit of MgCh. Silencing of *Brchli1* in Chinese cabbage plants resulted in a bright yellow leaves phenotype. Transiently overexpressed *Brchli1* in the *byl* mutant restored the green leaf phenotype and significantly increased relative *Brchli1* expression levels. Both BrCHLI1 and its mutated variant were localized in chloroplasts. Yeast two-hybrid and luciferase complementation imaging assays demonstrated that BrCHLI1 interacted with both BrCHLD and itself. BrCHLI1 mutations did not affect its interaction with BrCHLD, but impaired its interaction with itself. This study is the first to clone the *BrChli1* gene in Chinese cabbage, and the results provide insights into the molecular mechanism of leaf color formation.

## Materials and methods

### Plant materials

Wild-type ‘FT’ Chinese cabbage (*Brassica rapa* L. ssp. *pekinensis*) was obtained from microspore culture as a DH line (Huang et al., 2016). The *bright yellow leaves* (*byl*) mutant was obtained by immersing germinated seeds of ‘FT’ in EMS. Hybridization of *byl* with ‘FT’ resulted in the F_1_, F_2_, and BC_1_ generations for genetic analysis, with the F_2_ population also utilized for phenotypic identification, gene mapping, and KASP analysis.

### Determination of photosynthetic pigment content

Leaves of *byl* and ‘FT’ at 20 days after seeding (DAS) were individually sliced into small pieces weighing 0.1 g and placed into 10 mL of 80% (v/v) acetone for dark treatment until the leaves turned white. The absorbance values of the samples at wavelengths of 663 nm, 645 nm, and 470 nm were measured using a spectrophotometer. Levels of Chl a, Chl b, and carotenoids were calculated following previously reported methods (Holm, 1954).

### Measurement of photosynthesis and Chl fluorescence parameters

Ten plants each of *byl* and ‘FT’ (40 DAS) were selected on a sunny morning for photosynthetic parameter measurements. The sixth true leaf from each plant was used to measure photosynthetic characteristics using a Li-6800 instrument (LI-COR Biotechnology, USA). Light intensity was set to 1500 μmol m^-2^ s^-1^. After achieving a stable net photosynthetic rate, readings for net photosynthetic rate (*Pn*), transpiration rate (*Ts*), stomatal conductance (*Ls*), and intercellular CO_2_ concentration (*Ci*) were recorded.

Ten plants each of *byl* and ‘FT’ (40 DAS) were selected for Chl fluorescence parameter measurements as described previously (Zhao et al., 2022).

### Ultrastructural observation of chloroplast

Leaves of *byl* and ‘FT’ plants (40DAS) were sliced into 1 mm × 2 mm pieces. Sample handling was performed as described previously (Zhao et al., 2014). The ultrastructure of chloroplasts was observed using an H-7700 TEM instrument (Hitachi, Japan).

### Identification of the candidate gene

Identification of the candidate gene *Brchli1* was performed using an improved MutMap approach (Abe et al., 2012). The bright yellow leaves mutant pool was generated by pooling DNA from 70 F_2_ plants displaying the bright yellow leaves phenotype. DNA extraction was performed using a DNAsecure Plant Kit (Tiangen Biotech Ltd., Beijing, China) for ‘FT’ and the bright yellow leaves mutant pool. Subsequent resequencing was conducted using a NovaSeq 6000 sequencer (Illumina, San Diego, California, USA). High-quality data were obtained by filtering raw data using the sliding window method with fastp (v0.20.0) (Gao et al., 2020). High-quality filtered data were aligned to the reference genome using bwa (0.7.12-r1039) (Li and Durbin, 2010). GATK (McKenna et al., 2010) was utilized for the detection of SNPs and insertions and deletions (INDELs). Functional annotation was performed using ANNOVAR (Wang et al., 2010), and Circos (Krzywinski et al., 2009) was employed to map mutation information onto the genome.

### SNP genotyping by KASP

Genotype analysis was conducted using KASP to detect the co-segregation of SNPs, identifying candidate genes for *Brchli1.* The KASP experiment was conducted on 157 F_2_ individuals with the bright yellow leaves phenotype and two ‘FT’ plants. The KASP thermal cycling conditions were set as previously described (Xi et al., 2018).

### Cloning and sequencing of *Brchli1*

The DNA and coding sequences of the *Brchli1* gene from *byl* and ‘FT’ were amplified. The specific operational procedures were performed as described previously (Liu et al., 2021). Sequencing was performed at Sangon Biotech (Shanghai, China) using the Sanger method.

### Protein characterization and phylogenetic analysis of BrCHLI1

A phylogenetic tree was constructed using the neighbor-joining method with 1000 bootstrap replicates in MEGA6.0. Multiple sequence alignment of amino acid sequences was performed using DNAMAN v6.0. Protein structural domains of BrCHLI1 were predicted using SMART (https://smart.embl-Heidelberg.de/).

### VIGS analysis

To further investigate the function of *Brchli1* in response to golden Chinese cabbage leaves, a 300 bp Brchli1-specific segment was cloned into the viral vector pTRV2 following a previously described procedure (Pflieger et al., 2010). Next, pTRV2-*Brchli1*, pTRV2, and pTRV1 were individually transformed into *A. tumefaciens* GV3101 using the freeze-thaw method (Zhou et al., 2021). The GV3101 suspension containing pTRV1 was mixed in a 1:1 ratio with GV3101 suspensions containing pTRV2-*0* (empty vector) or pTRV2-*Brchli1*, and used to infect newly germinated ‘FT’ seeds. Each group infected 90 seeds (three replicates). Seeds were then sown in a greenhouse, and silencing efficiency was assessed 10, 20, and 30 days post-infection.

### Functional validation of transient overexpression

The coding sequence of *Brchli1* lacking the stop codon was inserted into the pSuper1300-GFP (pSuper::GFP) vector, and transformed into the *byl* mutant alongside the control group pSuper::GFP using the VIGS method. Each group of 90 seeds (three replicates), which were subsequently planted in a greenhouse. Transient overexpression efficiency was evaluated at 10, 20, and 30 days after infection.

### Subcellular localization of BrCHLI1 and *Brchli1*

The coding regions of the BrCHLI1 and Brchli1 sequences, excluding the stop codons, were cloned into the ProCAMV35S:BrCHLI1:GFP and ProCAMV35S:Brchli1:GFP vectors to determine the subcellular localization of the BrCHLI1 and Brchli1 proteins. After sequencing validation, vectors were transferred into *Agrobacterium tumefaciens* GV3101, and injected into tobacco leaf mesophyll cells at an OD_600_ of 0.6−0.8, followed by a 24 h dark treatment then a 24 h light treatment. The 35S-driven GFP vector served as a negative control. Fluorescence signals were observed using a confocal laser scanning microscope (Leica Microsystems, Wetzlar, Germany). The GFP fluorescence and emission signals of Chl autofluorescence were detected at wavelengths of 496−540 nm and 643−730 nm, respectively.

### Y2H assays

Y2H assays were conducted following the manufacturer’s instructions (Coolaber, Beijing, China). The full-length CDS of *Brchli1* was cloned into the pGBKT7 and pGADT7 vectors to obtain pGBKT7-BrCHLI1 and pGADT7-BrCHLI1. Point-mutated *Brmchli1* was cloned into the pGBKT7 vector to obtain pGBKT7-Brchli1. The full-length CDS of *Brchld* was cloned into the pGADT7 vector to obtain pGADT7-BrCHLD. pGBKT7-53 + pGADT7-T served as the positive control, while pGBKT7-Lam + pGADT7-T served as the negative control. pGBKT7-BrCHLI1 + pGADT7-BrCHLI1, pGBKT7-Brchli1 + pGADT7-BrCHLI1, pGBKT7-BrCHLI1 + pGADT7-BrMCHLD, and pGBKT7-Brchli1 + pGADT7-BrMCHLD were co-transformed into yeast competent cells (Y2HGold strain) and plated on SD/-Leu-Trp (DDO) solid medium. Positive clones were picked and transferred to SD/-Leu-Trp-His-Ade + 30 mg L^-1^ X-α-gal (QDO/X) solid medium.

### Luciferase complementation imaging assays

The full-length CDS of BrCHLI1 was incorporated into the N-terminal luciferase (pCAMBIA1300-nLUC) and C-terminal luciferase (pCAMBIA1300-cLUC) vectors. Full-length CDS of BrCHLD and Brchli1 were also incorporated into the N-terminal luciferase vector. nLUC and cLUC were co-injected into tobacco leaves, followed by dark treatment for 24 h and light treatment for another 24 h at 25°C. The leaves were coated with a solution of potassium luciferin and observed using a Night Shade LB 985 system (Berthold, Bad Wildbad, Germany).

### qRT-PCR

RNA extracted from the leaves of ‘FT’ was subsequently reverse-transcribed into cDNA. qRT-PCR was conducted using Ultra SYBR Green Mix (Kangwei Century, Beijing, China) and a QuantStudio 6 PCR system. *ACTIN* was employed as an internal control. Relative expression levels were calculated using the 2^-ΔΔCt^ method.

## Results

### Bright yellow leaves and impaired chloroplast development in *byl*

**Figure 1:**
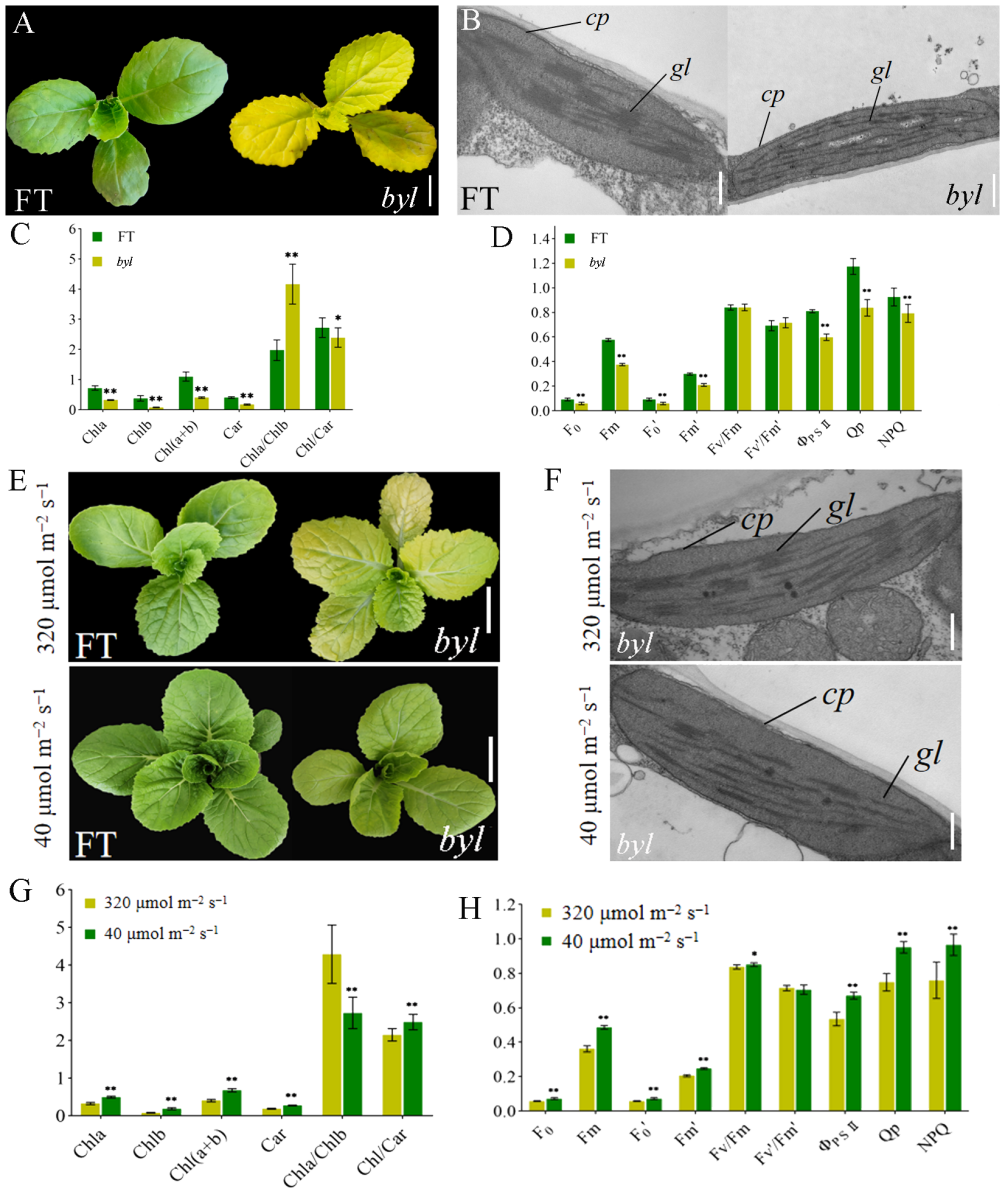
Morphological characteristics of ‘FT’ and *byl*. **(A)** Phenotypic observations of ‘FT’ and *byl* 20 days after sowing. Scale bar = 1 cm. **(B)** TEM observations of ‘FT’ and *byl*. Scale bar = 200 nm. Note: *cp* chloroplast, *gl* grana lamella. **(C)** Photosynthetic pigment content in ‘FT’ and *byl*. * represents significant differences at p < 0.05 (Student’s t-test), ** represents significant differences at p < 0.01. **(D)** Fluorescence dynamics parameters of ‘FT’ and *byl.* ** represents significant differences at p < 0.01. **(E)** Phenotypic observations of ‘FT’ and *byl* under varying light intensities. Scale bar = 1 cm. **(F)** TEM observations of ‘FT’ and *byl* under varying light intensities. Scale bar = 200 nm. *cp*, chloroplast; *gl*, grana lamella. * represents significant differences at p < 0.05 (Student’s t-test), **(G)** Photosynthetic pigment content in *byl* under varying light intensities. ** represents significant differences at p < 0.01. **(H)** Fluorescence dynamics parameters of *byl* under varying light intensities. * represents significant differences at p < 0.05 (Student’s t-test), ** represents significant differences at p < 0.01.

The *bright yellow leaves* (*byl*) mutant was obtained by EMS mutagenesis of DH line ‘FT’ seeds. Compared with the green leaves of ‘FT’, *byl* leaves were golden, with no impact on growth and development (**Figure 1A**). To investigate the effect of *byl* on chloroplast development, transmission electron microscopy (TEM) was used to observe chloroplasts in ‘FT’ and *byl* leaves, revealing fewer and sparser stacked layers of thylakoid grana in *byl* compared to the well-organized grana observed in ‘FT’ (**Figure 1B**). Levels of Chl*a*, Chl*b*, and Car in *byl* were significantly lower than in ‘FT’, suggesting a potential association between the bright yellow leaves phenotype of *byl* and insufficient Chl accumulation (**Figure 1C**). The *byl* mutant had a significantly lower net photosynthetic rate (*Pn*), stomatal conductance (*Gs*), and transpiration rate (*Ts*) than ‘FT’. In addition, the intercellular CO_2_ concentration (*Ci*) was higher in *byl* than in ‘FT’. These results suggest that the reduced photosynthetic pigment content in *byl* may have impaired *Pn*. The higher *Ci* in *byl* indicated a lower CO_2_ utilization efficiency, leading to a reduction in *Pn* (**Supplementary Table 1**). The values of minimum fluorescence under dark adaptation (F_0_), maximum fluorescence under dark adaptation (Fm), minimum fluorescence under light adaptation (F_0_’), maximum fluorescence under light adaptation (Fm’), actual photochemical efficiency of PSII (Φ_PSII_), photochemical quenching (Qp), and non-photochemical quenching (NPQ) in *byl* were significantly lower than in ‘FT’, consistent with the reduction in Chl content in *byl* leaves (**Figure 1D**).

Genetic analysis of the offspring of *byl* and ‘FT’ showed that leaves of the F_1_ generation plants were all green. In the F_2_ generation population, there were 571 green-leaved plants and 201 golden-leaved plants, conforming to a 3:1 ratio (χ^2^ = 0.44). This implies that the bright yellow leaves mutation trait in *byl* is controlled by a pair of recessive nuclear genes (**Supplementary Table 2**).

### *byl* turned light green in low light intensity

The *byl* mutant displayed distinct phenotypes under different light intensities, exhibiting bright yellow leaves in high light intensity (320 μmol m^−2^ s^−1^) and lighter green in low light intensity (40 μmol m^−2^ s^−1^). ‘FT’ and *byl* were cultured for 10 days under light intensities of 320 μmol m^−2^ s^−1^ and 40 μmol m^−2^ s^−1^, respectively; *byl* exhibited a change in leaf color, while ‘FT’ remained unaffected (**Figure 1E**). TEM revealed *byl* chloroplasts returning to normal under low light intensity (**Figure 1F**). At 40 μmol m^−2^ s^−1^ light intensity, there was a significant increase in levels of Chl*a*, Chl*b*, and Car in *byl* (**Figure 1G**), indicating an enhancement in photosynthetic pigment content under low light conditions. Moreover, *byl* exhibited significant increases in *Pn*, *Gs*, and *E* (**Supplementary Table 3**), along with significant elevations in F_0_, Fm, F_0_’, Fm’, Φ_PSII_, Qp, and NPQ (**Figure 1H**). The increase in photosynthetic pigment content under 40 μmol m^−2^ s^−1^ light intensity also enhanced the photosynthetic rate of *byl*.

### Identification of the candidate gene for *Brchli1*

**Figure 2:**
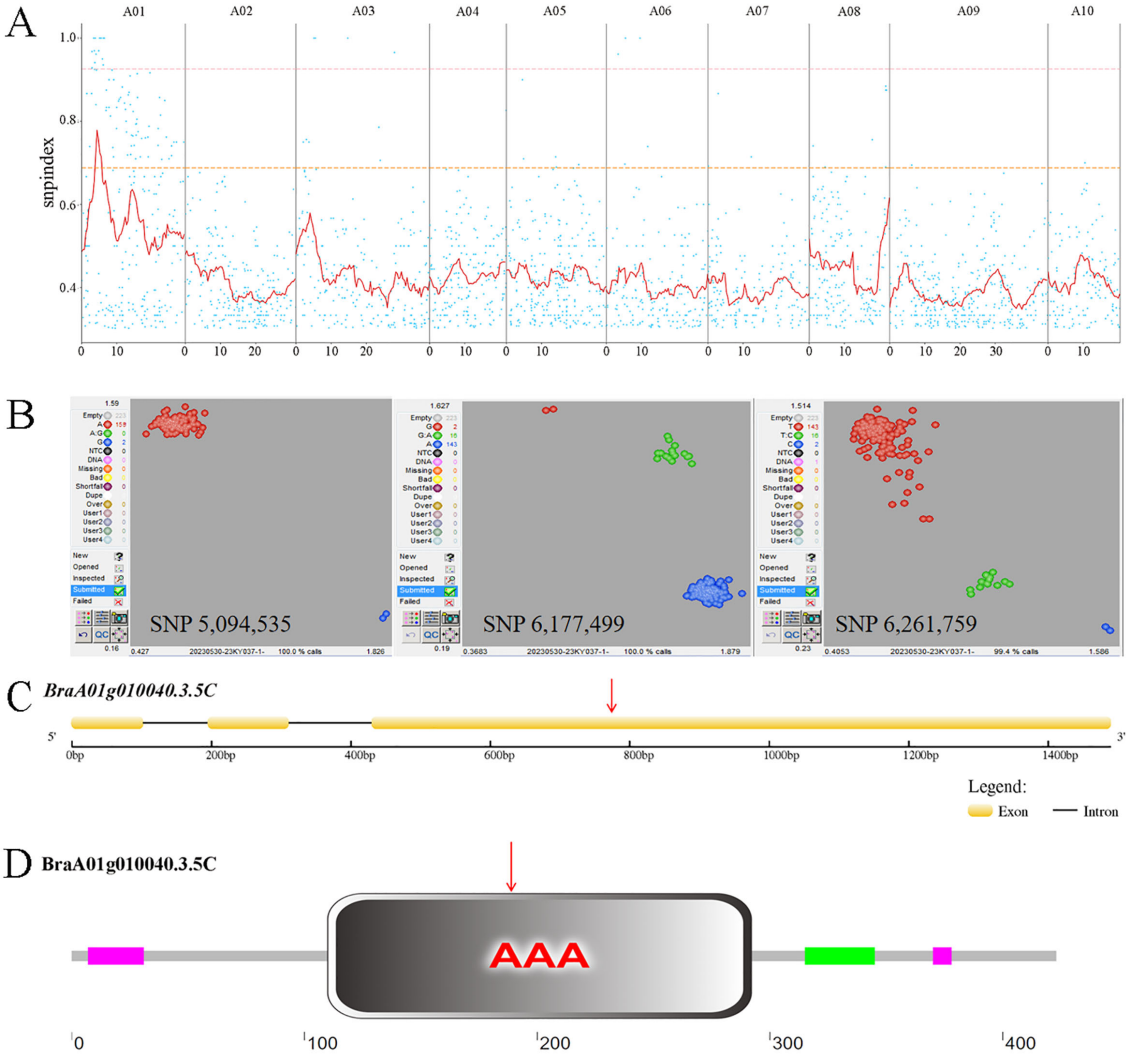
MutMap SNP index plot and KASP genotyping. **(A)** MutMap SNP index plot. Dots represent SNP-index values, the red line represents the mean SNP-index within the window, the pink line corresponds to the threshold line at the 99th percentile, and the orange line corresponds to the threshold line at the 95th percentile. **(B)** KASP genotyping of three candidate SNPs. In SNP 5,094,535, red dots represent the A:A genotype and blue dots represent the G:G genotype. In SNP 6,177,499, red dots represent the G:G genotype, green dots represent the G:A genotype, and blue dots represent the A:A genotype. In SNP 6,261,759, red dots represent the T:T genotype, green dots represent the T:C genotype, and blue dots represent the C:C genotype. **(C)** *Brchli1* gene structure. Red arrows indicate the mutation sites. **(D)** Domain distribution of BrCHLI1. Red arrows indicate the mutation sites.

We employed a modified MutMap method to identify the candidate gene. Via resequencing we obtained 175,935,758 (95.25%) and 162,498,262 (96.23%) HQ_Reads for ‘FT’ and the mutant pool, respectively, of which 93.06% and 93.07% were aligned to the Brara_Chiifu_V3.5 reference genome. Single-nucleotide polymorphisms (SNPs) between ‘FT’ and the mutant pool were detected and filtered, resulting in 366,300 SNPs located in exons. SNP-index was computed and filtered, followed by plotting its distribution across the chromosome (**Figure 2A**). Using a 0.95 SNP index as the threshold, we identified a candidate region of 5.6 Mb (2,000,000−7,600,000) on chromosome A01. Within the candidate interval, there were 58 SNPs, 12 of which were located in exonic regions, including three non-synonymous mutations (**Supplementary Table 4**).

To further validate the candidate SNPs, we conducted KASP genotyping on three non-synonymous mutation SNPs using a population of two ‘FT’ and 159 F_2_ generation individuals with the mutant phenotype. The results indicated that only SNP 5,094,535 within *BraA01g010040.3.5C* co-segregates with the mutant phenotype, with all mutants being A:A and both wild-type ‘FT’ plants being G:G. By contrast, both SNP 6,177,499 and SNP 6,261,759 exhibited recombinants, with both showing 16 G:A genotypes in mutant phenotype plants (**Figure 2B**). The homologous gene of *BraA01g010040.3.5C* in *Arabidopsis*, *AT4G18480*, encodes the CHLI subunit of Mgch, which is required for Chl biosynthesis. Therefore, we hypothesized that *BraA01g010040.3.5C* was a candidate gene for *Brchli1*.

### Cloning and sequence analysis of *Brchli1*

The full-length and coding sequence (CDS) of *BraA01g010040.3.5C* were cloned from ‘FT’ and *byl*, revealing that *BraA01g010040.3.5C* spans 1666 bp with a CDS of 1272 bp encoding 424 amino acids (**Figure 2C**). This gene consisted of three exons and two introns, with a G to A mutation at position 783 (**Supplementary Figure S1**) that changes the GAT codon to AAT (**Figure 2D**), causing substitution of aspartic acid (Asp) with asparagine (Asn). The mutation site of *Brchli1* is located on the conserved domain AAA of BrCHLI1.

### Functional verification of *Brchli1*

**Figure 3:**
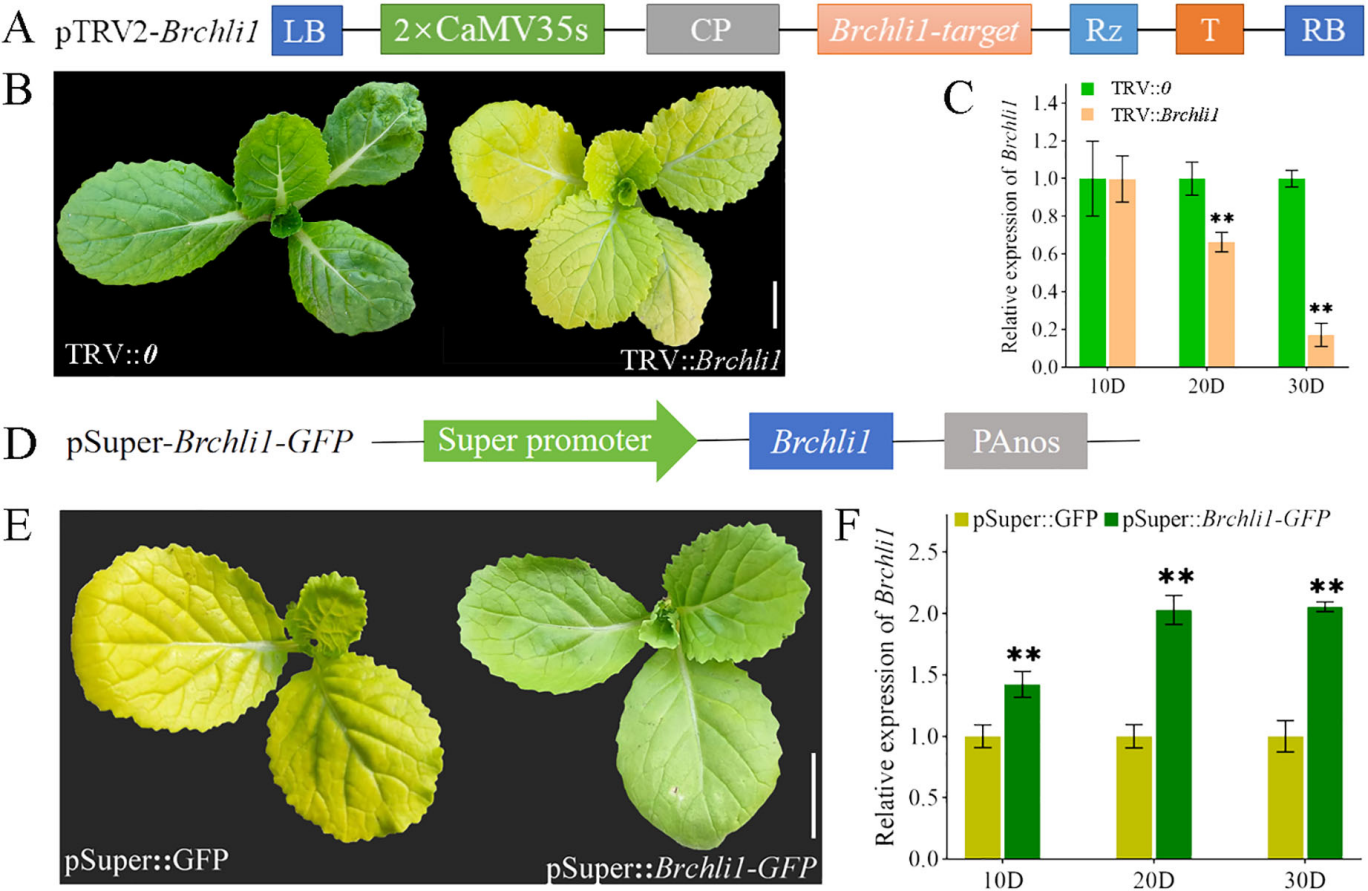
Silencing and transient overexpression of the *Brchli*. **(A)** Schematic diagram of carrier construction used in VIGS analysis. **(B)** Plant phenotypes of TRV::*0* and TRV::*Brchli1* at 30D. Scale bar = 1 cm. **(C)** Relative expression levels of *Brchli1* in TRV::*0* and TRV::*Brchli1* at 10, 20, and 30 days. ** represents significant differences at p < 0.01. **(D)** Schematic diagram of transient overexpression vector structure. **(E)** Plant phenotypes of pSuper: GFP and pSuper::*Brchli1-GFP* at 20D. Scale bar = 1 cm. **(F)** Relative expression levels of *Brchli1* in pSuper: GFP and pSuper::*Brchli1-GFP* at 10, 20, and 30 days. ** represents significant differences at p < 0.01.

To further confirm whether *Brchli1* was responsible for the bright yellow leaves phenotype of the *byl* mutant, a pTRV2-*Brchli1* recombinant vector was constructed (**Figure 3A**). We assessed gene silencing efficiency at 10, 20, and 30 days following virus-induced gene silencing (VIGS) treatment. Unlike the TRV::*0* group, the TRV::*Brchli1* silencing group displayed bright yellow leaves (**Figure 3B**). At 10 days post-treatment, there was no significant difference in *Brchli1* expression between TRV::*0* and TRV::*Brchli1*. By day 20, *Brchli1* expression was significantly decreased in TRV::*Brchli1* compared to the blank control, with the most pronounced decrease observed after 30 days (**Figure 3C**).

Additionally, we transiently overexpressed the vector pSuper::*Brchli1-GFP* in the mutant *byl* (**Figure 3D**). The results indicated that *byl* plant containing pSuper::*Brchli1-GFP* exhibited restored green leaf phenotype (**Figure 3E**), accompanied by a significant increase in relative expression levels of *Brchli1* (**Figure 3F**). These results implied that *Brchli1* is the causative factor for the bright yellow leaves of the *byl* mutant.

### Phylogenetic and structural analysis of BrCHLI1

**Figure 4:**
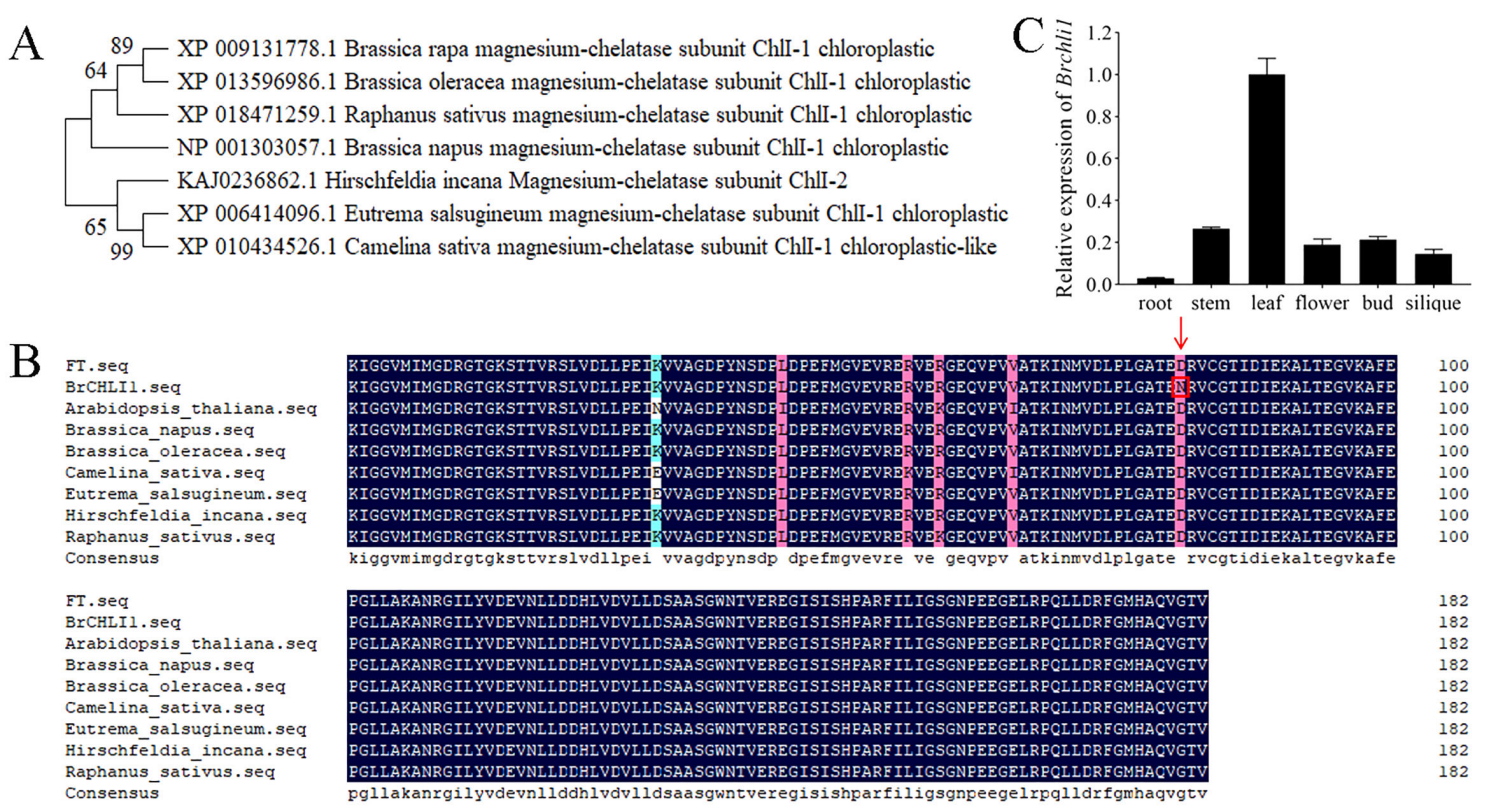
Phylogenetic tree of BrCHLI1, alignment of the AAA domain in BrCHLI1, and expression levels of *Brchli1*. **(A)** Phylogenetic analysis of BrCHLI1. **(B)** AAA sequence alignment. Black shaded regions represent 100% similarity, while pink shaded regions represent >75% similarity. Red arrows and red boxes denote the mutation sites. **(C)** Relative expression levels of *Brchli1* in different organs of ‘FT’.

The BrCHLI1 protein comprises 423 amino acids with a molecular weight of 45.9 kDa and a theoretical isoelectric point of 6.41. A phylogenetic tree was constructed to elucidate the evolutionary relationships between BrCHLI1 homologous sequences in different species, revealing high homology among various crops of the *Brassicaceae* family (**Figure 4A**). The AAA domain of the BrCHLI1 protein is highly conserved across *Cruciferae* species (**Figure 4B**).

### *Brchli1* exhibits high expression in leaves

We quantified the relative transcription levels of *Brchli1* across diverse plant organs using quantitative reverse transcription PCR (qRT-PCR) to explore its physiological functionality. In ‘FT’, expression of *Brchli1* was highest in leaves, followed sequentially by stems, buds, flowers, and siliques, with lowest expression in roots (**Figure 4C**). Hence, *Brchli1* plays a significant role in Chinese cabbage leaves.

### Subcellular localization of BrCHLI1 and *Brchli1* within chloroplasts

**Figure 5:**
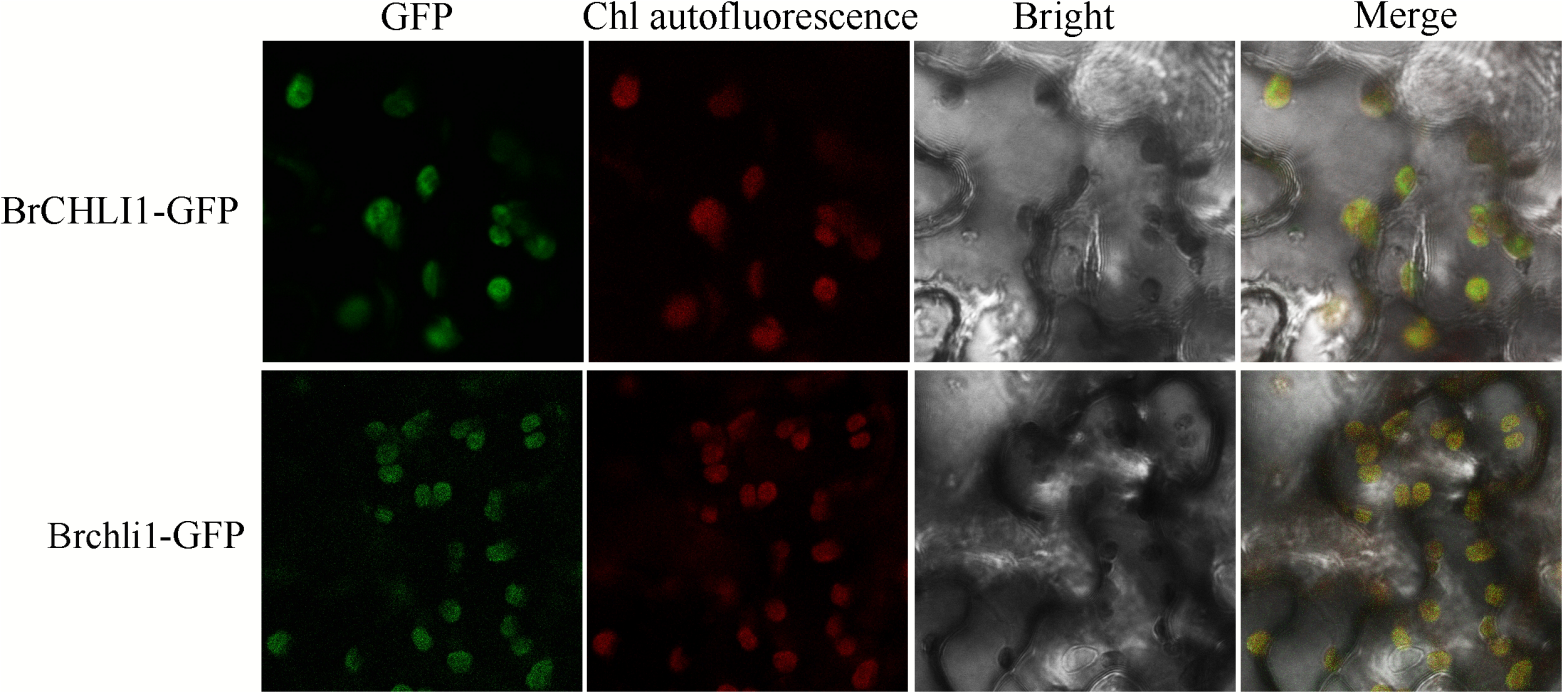
Subcellular localization of BrCHLI1 and Brchli1.

To ascertain whether the mutation in BrCHLI1 affected its expression localization, BrCHLI1-green fluorescent protein (GFP) and the mutated Brchli1-GFP were introduced into tobacco, and their co-localization with chloroplast autofluorescence was assessed. The results demonstrated that the GFP fluorescence of both BrCHLI1 and Brchli1 was localized within chloroplasts (**Figure 5**), with the mutation in BrCHLI1 having no effect on the localization.

### The mutation in BrCHLI1 does not affect interaction with BrCHLD

**Figure 6:**
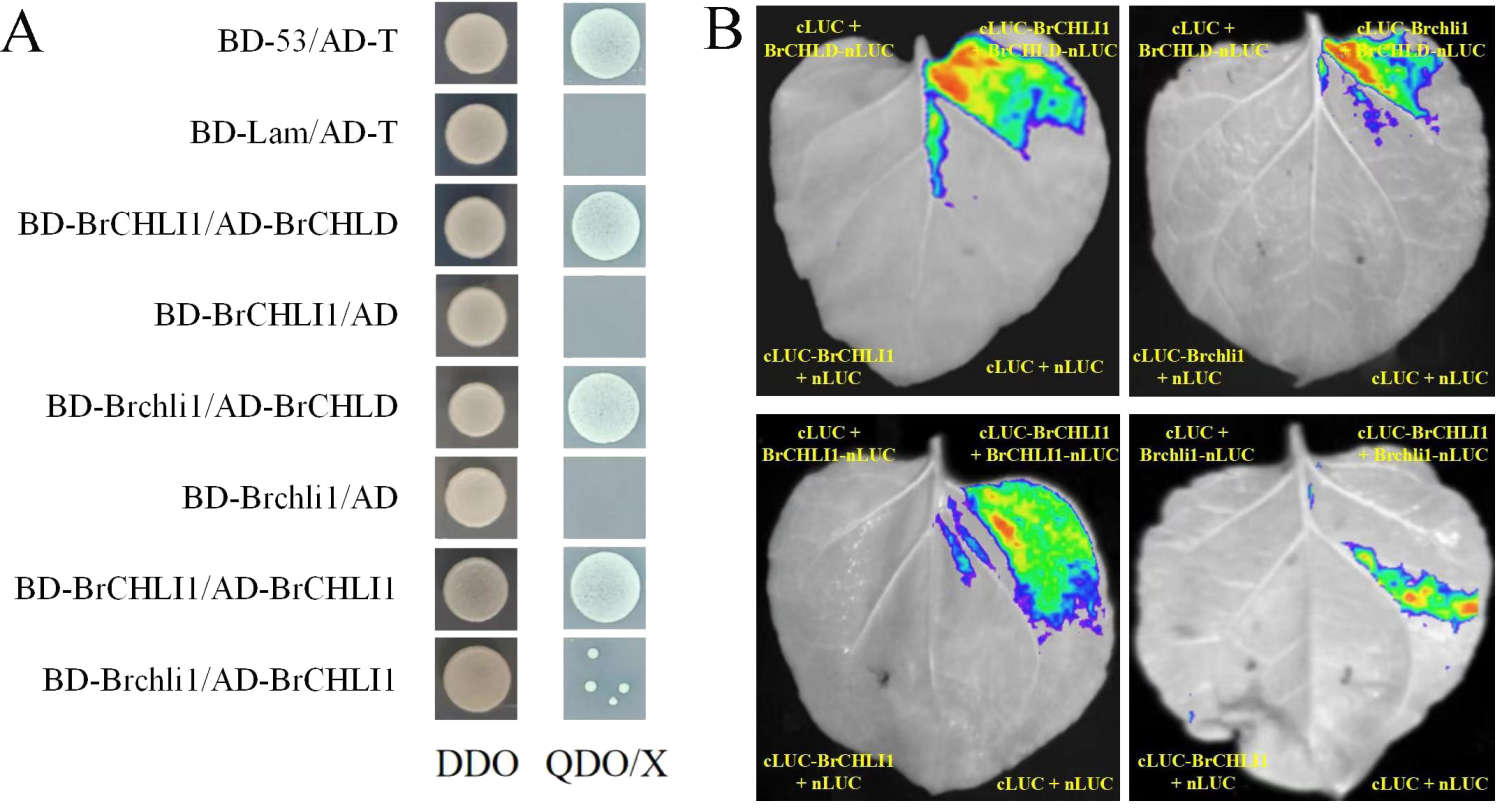
Interaction relationships between BrCHLI1 and Brchli1 with BrCHLD and BrCHLI1. **(A)** Interaction relationships analyzed by Y2H. DDO, SD/-Leu-Trp. QDO/X, SD/-Leu-Trp. **(B)** Analysis of interplay/interaction using LCI in tobacco leaves.

To investigate the role of BrCHLI1 in Chinese cabbage leaf coloration and assess its interaction with BrCHLD, we co-transformed recombinant vectors pGBKT7-BrCHLI1 and pGADT7-BrCHLD into yeast cells using the yeast 2-hybrid (Y2H) system for interaction analysis. On the SD/−Trp/−Leu (DDO) plate, yeast colonies for pGBKT7-BrCHLI1 + pGADT7-BrCHLD, pGADT7-T + pGBKT7-53 (positive control), and pGADT7-T + pGBKT7-Lam (negative control) exhibited normal growth, confirming successful yeast transformation. PGBKT7-BrCHLI1 + PGADT7-BrCHLD, as well as the positive control, showed normal yeast growth on SD/-Leu-Trp-His-Ade-X-α-gal (QDO/X) plates, while the negative control did not exhibit any growth, indicating that BrCHLI1 interacted with BrCHLD (**Figure 6A**). To confirm this interaction *in vivo*, we conducted luciferase complementation imaging (LCI) analysis in tobacco. When co-expressing cLUC-BrCHLI1 and BrCHLD-nLUC, a strong luminescence signal was observed in leaves, while the control group containing empty vectors showed no luminescence signal, indicating that BrCHLI1 interacts with BrCHLD (**Figure 6B**). These results suggest that BrCHLI1 and BrCHLD interact both *in vivo* and *in vitro*.

To ascertain if the mutation in BrCHLI1 affected its interaction with BrCHLD, we employed Y2H assay to determine the relationship between Brchli1 and BrCHLD. Yeast cells co-transformed with pGBKT7-Brchli1 + pGADT7-BrCHLD grew on QDO/X plates, demonstrating that Brchli1 interacted with BrCHLD (**Figure 6A**). Co-transformation of cLUC-Brchli1 and BrCHLD-nLUC into tobacco leaves resulted in a strong fluorescence signal, confirming that Brchli1 interacted with BrCHLD *in vivo* (**Figure 6B**). Thus, the mutation in BrCHLI1 did not affect its interaction with BrCHLD.

### The BrCHLI1 mutation weakens self-interaction

To further explore the potential self-interaction of BrCHLI1, we conducted Y2H analysis and LCI assays. Yeast cells co-transformed with pGBKT7-BrCHLI1 and pGADT7-BrCHLI1 displayed normal growth on QDO/X plates, confirming that BrCHLI1 can bind by themselves (**Figure 6A**), and the LCI assay further validated this interaction. Bioluminescence signals were detected in plant cells co-expressing cLUC-BrCHLI1 and BrCHLI1-nLUC fusion proteins (**Figure 6B**), indicating successful reconstitution of luciferase activity due to their physical interaction. Control experiments with non-interacting protein pairs did not exhibit any significant bioluminescence, confirming the specificity of the observed signal.

Subsequently, the interaction between BrCHLI1 and Brchli1 was further determined. Y2H assays revealed a reduced yeast colony viability in co-expressing colonies of BrCHLI1 and Brchli1 (**Figure 6A**), indicating a weakened physical interaction between the proteins. This observation was further substantiated by LCI, in which only faint bioluminescence signals were detected in cells expressing BrCHLI1 and Brchli1 fusion proteins (**Figure 6B**). These results demonstrated a diminished self-interaction capability of BrCHLI1 following mutation.

## Discussion

In this study, we identified the *bright yellow leaves* (*byl)* mutant in Chinese cabbage, and cloned the mutant gene *BraA01g010140.3.5C*. Silencing of *BraA01g010140.3.5C* in plants resulted in a bright yellow leaves phenotype. Transiently overexpressed *Brchli1* in the *byl* mutant restored the green leaf phenotype. Chloroplast development of *byl* was impaired, with decreased Chl content, *Pn*, and NPQ. The *byl* mutant displayed photosensitive phenotypes under variable light intensities. Mutation in BrCHLI1 weakened its self-interaction.

The CHLI subunit is essential for catalytic activity of MgCh. CHLI is a member of the AAA+ protein family possessing ATP hydrolysis activity (Fodje et al., 2001). The chelation reaction catalyzed by MgCh is a highly ATP-dependent process (Palmer and Mascia, 1980). Chelation of Mg^2+^ and Proto IX catalyzed by MgCh is an important regulatory step in the Chl biosynthesis pathway (Jensen et al., 1996). Previous studies demonstrated that mutations in subunit I can impact Chl synthesis within plant leaves. Mutations in the *OsCHLI* gene of rice led to yellow leaves and a significant decrease in Chl content (Zhang et al., 2015). Mutations in the *AtCHLI1* (Koncz et al., 1990) and *CHLI2* genes in *Arabidopsis* both resulted in a pale green leaf phenotype (Kobayashi et al., 2008). Amino acid missense mutations in *ChlI* resulted in a yellow-green phenotype in rice (Zhang et al., 2006). *TaCHLI* single-nucleotide mutations in wheat were responsible for pale green leaves caused by impaired Chl synthesis (Wang et al., 2020). We identified a point mutation in *Brchli1* at position 783 in the *byl* mutant of Chinese cabbage, resulting in the substitution of Asp to Asn, which in turn resulted in bright yellow leaves phenotype. Phylogenetic analysis of the *CHLI* gene indicated a high degree of conservation across different species. The *Brchli1* gene was cloned from Chinese cabbage for the first time in this study.

In the Chl synthesis pathway, Mgch catalyzes the reaction of Proto IX to form Mg-proto IX, a critical regulatory point in the process (Masuda, 2008). In the rice yellow leaf mutant *ell*, loss of function of Mgch prevents the conversion of Proto IX to Mg-proto IX, leading to accumulation of Proto IX, and levels of Proto IX in the mutant were significantly increased (Zhang et al., 2015). In the wheat pale green leaf mutant *byl*, levels of Proto IX and Mg-proto IX were significantly lower than those in wild-type plants (Wang et al., 2020). In the strawberry pale green leaf mutant *CHLI*, the content of Mg-proto IX was significantly lower than that in wild-type plants (Ma et al., 2023).

In the presence of Mg^2+^ and ATP, the CHLI and CHLD subunits formed an AAA+ complex which subsequently interacted with the CHLH chelating subunit, resulting in the formation of an active chelatase (Farmer et al., 2019). ChlI protein complexes that self-assemble are known to contain 6−8 ChlI subunits (Jensen et al., 1998). A single-nucleotide mutation in subunit I of the rice yellow leaf mutant *ell* disrupted its interaction with the H subunit, leading to the loss of MgCh catalytic activity and appearance of the yellow leaf phenotype (Zhang et al., 2015). In the wheat yellow leaf mutant *chli*, the mutant protein Tachli-7A was unable to interact with TaCHLI-7A in yeast, but showed weak interaction in bimolecular fluorescence complementation assays (Wang et al., 2020). In the present study, Y2H and LCI assays showed that BrCHLI1 mutations did not affect its interaction with BrCHLD, but impaired its interaction with itself. An amino acid mutation in the conserved structural domain of subunit I in the *byl* mutant compromised the activity of the BrCHLI protein, and disrupted its protein-protein interaction with itself. As a result, the synthesis of Chl was impaired, resulting in the yellow leaf phenotype.

The *byl* mutant could convert from bright yellow leaves to light green leaves in low light intensity, and chloroplasts recovered to normal, suggesting that *byl* may be better adapted to low light conditions. Under low light conditions, the *Arabidopsis immutans* mutant retained green leaves, while it displayed variegated leaves under high light conditions (Rosso et al., 2009). Compared with lower light conditions, the Chl-less barley mutant NYB displayed a more evident yellow leaf color under high light intensities (Yuan et al., 2010b). The same phenotype has been reported for the *Petunia hybrida* yellow-green leaf mutant (Colijn et al., 1983), golden-leaf privet (Yuan et al., 2010a), and *Acer palmatum* ‘Jingling Huangfeng’ (Li et al., 2015). Therefore, the Chinese cabbage *byl* mutant provides a useful material for studying the potential molecular mechanisms underlying the relationship between light conditions, leaf color, and photosynthetic capacity.

Our study demonstrated that mutation in *Brchli1* led to disrupted chloroplast development and a reduction in Brchli1 self-interaction, ultimately resulting in the manifestation of a golden yellow leaf phenotype. The *chli1* mutant exhibited a transformation of leaf color to pale green under weak light conditions. These findings provide insights into the leaf color formation mechanism in Chinese cabbage.

## Data Availability

The original data generated in the study are included in the article/**Supplementary Material**. The MutMap sequencing datasets are available in the Sequence Read Archives (SRA) of the NCBI under BioProject ID: PRJNA1074262. Genomic sequences and gene annotation information of B.rapa are downloaded online at http://brassicadb.cn.

## Supplementary material

Supplementary Figure 1 Cloning of the *Brchli1* gene from ‘FT’ and *byl*. The mutation site is located in the red box.
