# Supplementary figures and images for: Brchli1 mutation induces bright yellow leaves by disrupting magnesium chelatase I subunit function in Chinese cabbage (Brassica rapa L. ssp. pekinensis)
Source: Front Plant Sci. 2024 Aug 30;15:1450242. doi: 10.3389/fpls.2024.1450242 (PMC11392721; doi:10.3389/fpls.2024.1450242)

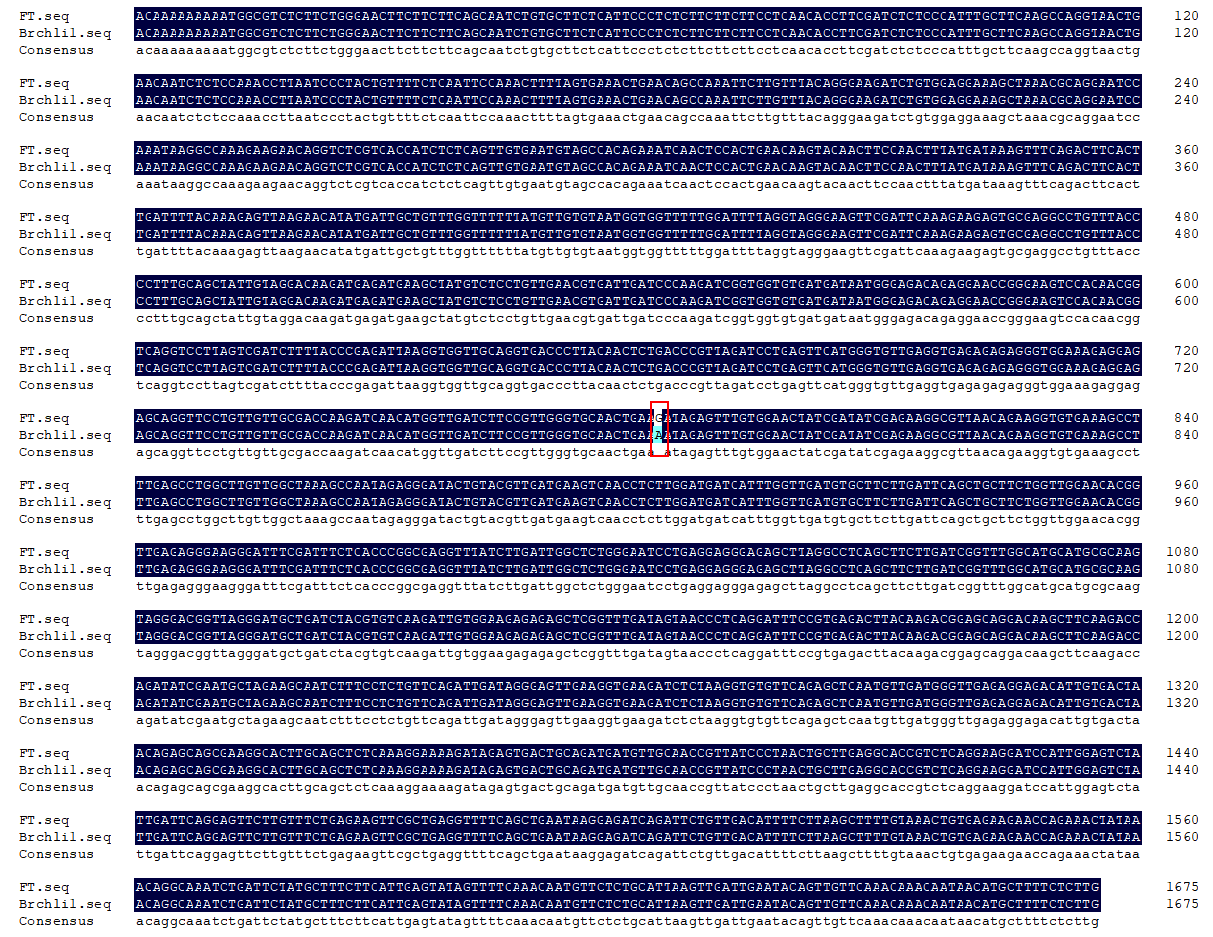

Supplement: Supplementary Figure 1 — Cloning of the Brchli1 gene from ‘FT’ and byl. The mutation site is located in the red box. [file Image1.tif]
